# Effect of Short-Term Desiccation, Recovery Time, and CAPA–PVK Neuropeptide on the Immune System of the Burying Beetle *Nicrophorus vespilloides*

**DOI:** 10.3389/fphys.2021.671463

**Published:** 2021-06-21

**Authors:** Arkadiusz Urbański, Karolina Walkowiak-Nowicka, Grzegorz Nowicki, Szymon Chowański, Grzegorz Rosiński

**Affiliations:** ^1^Department of Animal Physiology and Developmental Biology, Faculty of Biology, Adam Mickiewicz University in Poznań, Poznań, Poland; ^2^HiProMine S.A., Robakowo, Poland; ^3^Molecular Virology Research Unit, Faculty of Biology, Adam Mickiewicz University in Poznań, Poznań, Poland; ^4^genXone S.A., Złotniki, Poland

**Keywords:** burying beetles, stress conditions, neuropeptides, insect physiology, recovery

## Abstract

Environmental conditions, especially related to winter, are crucial for shaping activity of insect immune system. However, our previous research clearly indicates differences in the immune system functioning when the cold stress was induced in the laboratory conditions and when the beetles were collected from natural environment during winter. This is probably related to the multiplication of observed effects by simultaneous presence of different stress factors characteristic of winter, including desiccation. For these reasons, our next step was analysis of the effects of short-term desiccation and recovery time on the functioning of immune system of burying beetle *Nicrophorus vespilloides*. Also, the effect of Tenmo–PVK-2 (tenebrionid periviscerokinin), member of the CAPA–PVK neuropeptide family, was investigated to better understand observed changes. Short-term desiccation decreases the phagocytic activity of burying beetle haemocytes, which is correlated with a reduction in their adhesive ability. On the other hand, there was a significant increase in phenoloxidase (PO) activity and the level of *proPO* expression, which may suggest sealing the cuticula by melanin deposition and prevention of water loss. Additionally, the elevated level of *defensin* expression may be associated with the cross-talk between mechanisms, which participate in insect response to environmental stress, including pathogen infection. After 1 h of recovery time, the activity of tested cellular and humoral mechanisms was mostly back to the control level. However, inhibition of the activity of PO and down-regulation of *proPO* were noted. These results also indicate importance of melanin deposition during water loss. Moreover, it suggests that some changes in immune system functioning during stress conditions do not have an immune function. Interestingly, part of the effects characteristic of recovery time were also observed after the application of Tenmo–PVK-2, mainly related to haemocyte morphology. These results indicate that CAPA–PVK neuropeptides may also influence on activity of burying beetle immune system. It should be also highlighted that, because of the study of the effects of CAPA–PVK neuropeptides, homologs of vertebrate neuromedin U, the results may be interesting for search evolutionary similarities in the functioning of the neuroendocrine system of insects and vertebrates.

## Introduction

The activity of the insect immune system depends on many environmental factors, especially those related to atmospheric conditions, such as temperature and humidity (Catalán et al., 2012; Sinclair et al., 2013). However, there are still gaps in knowledge particularly regarding the impact of these stressors on the insect immune system. Our previous studies on the influence of environmental factors on the immune system activity of burying beetles showed that immune system activity of burying beetles changes differently when the stress factor (low temperature) was induced in laboratory conditions and when the individuals were exposed to low temperature in natural conditions (Urbański et al., 2017). This is probably related to the multiplication of the observed effects by simultaneous presence, in natural conditions, different stress factors characteristic of winter, including desiccation. For this reason, as the next step and the main aim of the presented research was evaluation of the effect of low air humidity on various immune mechanisms of burying beetles. Because of the current knowledge concerning the influence of recovery time after stress treatment on the insect physiology, this research variant was also included in the presented study.

Insect response to desiccation stress is inseparably linked to hormonal regulation *via* CAPA–PVK neuropeptide signaling (Terhzaz et al., 2015; MacMillan et al., 2018; Lubawy et al., 2020). The first identified member of insect CAPA–PVK neuropeptides was Manse-CAP_2b_^1^ (cardioacceleratory peptide 2b) isolated from the ventral nerve cord of the *Manduca sexta* moth (Huesmann et al., 1995). Its name comes from the cardioacceleratory properties and stimulation of contraction of visceral muscles. However, previous studies on function of CAPA–PVK neuropeptides showed that these neurohormones are primarily involved in regulation of insect diuresis (Predel and Wegener, 2006). It is especially visible in the first hours of recovery time after desiccation. Research conducted by Terhzaz et al. (2015) showed that the concentration of the CAPA–PVK neuropeptide precursor increases during desiccation and that these neuropeptides begin to be released from the nervous tissue during the recovery time just after the end of low-humidity treatment. Despite the fact that CAPA–PVK neuropeptides participate in the regulation of ion–water balance, there is also some evidence that these peptides can influence the immune system activity. The results obtained by Terhzaz et al. (2014) showed that Drome–CAPA–1, in Malpighian tubules of the fruit fly *Drosophila melanogaster*, stimulated the expression of *relish*, gene encoding one of the major components of Imd (immune deficiency) signaling pathway. Further research demonstrated that Drome–CAPA–1 in a similar manner to bacterial peptidoglycan, induced activation, and nuclear translocation of Relish protein in Malpighian tubules of *D. melanogaster*. But this did not lead to the expression of the gene that encodes diptericin, one of the major antimicrobial peptides (AMPs) in Diptera (Terhzaz et al., 2014). In addition, the structural and functional homology of CAPA–PVK neuropeptides and vertebrate neuromedin U (NMU) might also suggest that CAPA–PVK neuropeptides could influence the regulation of the activity of the insect immune system. Research by Terhzaz et al. (2012) showed that vertebrate NMU is a putative functional homolog of Drome–CAPA–1 and −2. For example, authors proved that NMU-25 increases fluid transport by the Malpighian tubule in *D. melanogaster*. Also, the functional homology between these two neuropeptide families is related to the fact that CAPA–PVK neuropeptides and NMU participate in regulation of contractile activity of visceral muscles (Brighton et al., 2004; Budhiraja and Chugh, 2009; Chowański et al., 2016). Moreover, both neuropeptide families are involved in regulation of ion transport (Huesmann et al., 1995; Brighton et al., 2004; Predel and Wegener, 2006). Because of these, a possibility that CAPA–PVK neuropeptides, similar to NMU, may participate in regulation of immune system functioning exists.

The role of NMU in the functioning of the vertebrate immune system is mainly related to the cellular response. This neuropeptide has a dose-dependent stimulating effect on cell adhesion, chemotaxis, and promotion of eosinophil infiltration into inflammatory sites (Moriyama et al., 2006; Kono et al., 2012). For these reasons, it is possible that administration of CAPA–PVK neuropeptides leads to changes in the cellular response also in insects. Because of that, an equally important part of the presented research is the analysis of the influence of the CAPA–PVK neuropeptide on the activity of the immune system of *Nicrophorus vespilloides*. The effect of the peptide administration was tested 1 h after injection to simulate endogenous release of the tested neuropeptide after 1-h recovery time. The potential similarity of the results in variant with 1-h recovery and the results obtained in case of beetles injected with CAPA–PVK neuropeptide may suggest that the observed effects after recovery time may be closely related to the action of tested neuropeptide.

## Materials and Methods

### Insects

Adult *N. vespilloides* were obtained from a culture maintained at the Department of Animal Physiology and Department of Animal Physiology and Developmental Biology at Adam Mickiewicz University in Poznań, according to the methods described previously by Urbański et al. (2017). Beetles were raised under conditions that simulated summer in northern Poland [15 h of light and 9 h of dark, 21°C and approximately 80% of relative humidity (RH)]. In the studies, we used only 7-day-old adults from the F1 generation. Individuals were maintained in separate plastic containers filled with moist soil and fed with cat food twice a week. The parents were captured in a natural environment located near Poznań (Suchy Las, 52°28′38.92′′N, 16°53′56.71′′E). Reproduction of burying beetles was induced by providing a male–female pair with adult mouse carcass (approximately 35 g each, commercially available livestock feeding mouse).

### Desiccation Treatment of Beetles

A glass desiccator filled with silica beads (Sigma–Aldrich) was used to expose the burying beetles to low humidity. The RH in the chamber fluctuated within 10–15%. The volume of silica beads was experimentally adjusted to obtain specific humidity. Each beetle was placed in the desiccator in a separate plastic container for 12 h. Inside the desiccators, the conditions were monitored by TFA Dostmann KlimaLogg Pro thermohygrometers (accuracy: temperature: ± 1°C and RH: ± 3%; resolution: temperature: 0.1°C and RH: 1%). During the experiment, the previously mentioned photoperiod was maintained. For a short recovery examination, after low-humidity treatment, they were returned to the plastic containers with moist soil for 1 h. The measurements were performed just after treatment and 1 h after treatment (recovery variant). To facilitate the presentation of the results, the names of research treatments have been simplified. The abbreviations used in this article are defined below.

LH12: Exposure to low humidity for 12 h.

LH12R1: Exposure to low humidity for 12 h and recovery for 1 h.

The controls for these groups were untreated burying beetles.

The duration of low-humidity treatment was chosen based on the results of research conducted by Bedick et al. (2006) and our preliminary studies. The results showed that after 12 h of low-humidity treatment, burying beetles were characterized by high water loss (Bedick et al., 2006). Our observation also confirmed these results (see section “Weight Loss”).

### Weight Loss

To confirm the effect of low humidity on water loss by burying beetles, the weight of tested individuals was checked just before and just after 12-h low-humidity treatment. Beetles were weighed using Mettler Toledo ML54 scale (accuracy 0.1 mg). For reduction of errors, each measurement was repeated twice. The individual weight was an average value of these two measurements. In the experiment, 12 individuals were used.

### Neuropeptide Solutions Used

Aside from those mentioned in the section desiccation treatment of beetles, part of the tested burying beetles was injected with Tenmo–PVK-2 (RIGKMVSFPRIa; *Tenebrio molitor* periviscerokinin-2, AN-DRX001789) solution, one of the members of CAPA–PVK neuropeptides family (Neupert et al., 2018; Supplementary Figure 1). In the presented study, two neuropeptide concentrations were tested, 10^–9^ and 10^–5^ M (the final concentration in the insect body is at least 10 times lower: 10^–10^ and 10^–6^ M, respectively). The neuropeptide was synthesized by Creative Peptides (Shirley, NY, United States; purity >95% high-performance liquid chromatography). Two microliters of neuropeptide solution with physiological saline appropriate for beetles (PS, 274 mM NaCl, 19 mM KCl, 9 mM CaCl_2_) was injected 1 h before analysis to simulated releasing of CAPA–PVK neuropeptide after recovery time. Control individuals were injected with 2 μL of PS.

### Injection and Haemolymph Collection

Before neuropeptide injection or hemolymph collection, beetles were anesthetized with CO_2_. Tenmo–PVK-2 was injected under the coxa of the third pair of legs using a microliter syringe (Hamilton). The hemolymph was collected by cutting the edge of the pronotum.

### Total Haemocyte Count Assay

The total haemocyte count (THC) was determined according to the modified method described by Urbański et al. (2017). In this assay, 2 μL of hemolymph was mixed with 20 μL of PS and an anticoagulation buffer (4.5 mM citric acid and 9 mM sodium citrate, 5:1 v/v). The THC was then determined using a Bürker chamber and a compound light microscope Zeiss Primo Star equipped with Axiocam 105 digital camera. In each repetition, 16 randomly selected fields of the Bürker chamber were analyzed and counted using ImageJ (version 2) software. At least seven individuals were used in each of the research treatments [control: 13; LH12: 7; LH12R1: 7; PS (control individuals injected with PS): 8; PVK 10^–9^ (individuals injected with PVK-2 in concentration 10^–9^ M): 7; PVK 10^–5^ (individuals injected with PVK-2 in concentration 10^–5^ M): 8].

### Haemocyte Morphology and Their Surface Area

The morphology of burying beetle haemocytes was analyzed according to the method described by Urbański et al. (2018). The method is based on an evaluation of the structure of the F-actin cytoskeleton and the nucleus of the haemocytes. First, a 2-μL sample of burying beetle hemolymph was gently mixed with 20 μL of physiological saline and an anticoagulation buffer. Then, 20 μL of this suspension was placed on a microscope slide precoated with poly-L-lysine (Sigma–Aldrich P4707) and incubated for 30 min at room temperature. The excess suspension was then removed, and the cover glass was washed with physiological saline. The haemocytes were subsequently fixed with 4% paraformaldehyde for 10 min, and the specimen was permeabilized with 0.1% Triton-X 100 (Sigma–Aldrich T8532). Next, the F-actin cytoskeleton of the haemocyte was stained with Oregon Green^®^ 488 phalloidin (Invitrogen) for 20 min in the dark at room temperature. The nuclei of the haemocytes were stained with a solution of DAPI in physiological saline (1:50 vol/vol). After the staining, the specimens were mounted with a mounting medium (90% glycerol with 2.5% DABCO). The morphology of the haemocytes was analyzed using a Nikon Eclipse TE 2000-U fluorescence microscope. Photographs were taken with a Nikon DS-1QM camera.

The adhesion of haemocytes was tested according to the method described by Urbański et al. (2018). This analysis is based on a measurement of the surface area of the haemocytes using AxioVision software (version 4.9.1). Estimation of haemocyte surface area after the equal incubation time allows to analyze adhesion and spreading abilities of tested cells. For photograph calibration, an algorithm implemented to AxioVision software was used. To minimize the individual variation between insects, treatment groups were compared to the control samples performed on the same day. At least five individuals (251 haemocytes) were analyzed for each treatment [control: 10 (817 cells); LH12: 5 (273 cells); LH12R1: 7 (299 cells); PS (control individuals injected with PS): 5 (251 cells); PVK 10^–9^ (individuals injected with PVK-2 in concentration 10^–9^ M): 8 (276 cells); PVK 10^–5^ (individuals injected with PVK-2 in concentration 10^–5^ M): 7 (373 cells)].

Based on the haemocyte morphology and characteristic features described by Urbański et al. (2017), the proportion of haemocytes belonging to different fractions was determined. For these measurements, the specimens were analyzed under light and fluorescence microscopy. The results are expressed as the average proportion of each type of haemocyte compared with the total number of haemocytes visible in the photographs.

### Percentage Ratio of Phagocytosis

The ability of burying beetle haemocytes to phagocytosis was tested under *in vitro* conditions based on the method described by Urbański et al. (2017). For this assay, a 2-μL sample of the hemolymph was incubated on a microscope slide with poly-L-lysine for 30 min at room temperature, together with a suspension of latex beads and physiological saline with an anticoagulation buffer (600:1 v/v). Excess suspension was removed, and the specimen was washed with physiological saline. After washing, the haemocytes were fixed with a 4% paraformaldehyde solution for 10 min. To visualize the haemocyte nuclei, a solution of DAPI and physiological saline (1:50 v/v) was then applied. Next, the specimens were mounted with mounting medium (90% glycerol with 2.5% DABCO) and analyzed using light and fluorescence microscopy. The proportion of phagocytic haemocytes was determined by comparing the number of haemocytes with fully phagocytized latex beads to the total number of haemocytes visible in the micrograph. At least 10 individuals were analyzed for each treatment [control: 16 (3,735 cells); LH12: 10 (2,224 cells); LH12R1: 12 (6,151 cells); PS (control individuals injected with PS): 10 (1,468 cells); PVK 10^–9^ (individuals injected with PVK-2 in concentration 10^–9^ M): 11 (1,943 cells); PVK 10^–5^ (individuals injected with PVK-2 in concentration 10^–5^ M): 10 (1,362 cells)]. For better visualization of differences between tested groups, the results are presented as the percentage change in phagocytosis ratio compared to control individuals. To minimize the individual variation between insects, treatment groups were compared to the control samples performed on the same day.

### Phenoloxidase Activity

The level of phenoloxidase (PO) activity was determined using the method previously described by Sorrentino et al. (2002) and Urbański et al. (2014). During the experiment, a sample of hemolymph (1 μL) was placed on a piece of white filter paper (Whatman no. 52) soaked in 10 mM phosphate buffer with L-DOPA (2 mg/mL). The samples were then incubated for 30 min at room temperature. Next, the filter paper was air-dried and scanned with a SHARP AR 153EN scanner (600 dpi, 8 bits, gray scale). The results were expressed as the mean pixel value measured at the center of each sample using ImageJ software (version 2) and are presented as the percent change in PO activity compared with the control individuals. To minimize the individual variation between insects, control samples were performed on the same day as treatment groups. At least 10 individuals were analyzed for each treatment. Two technical repetitions were made for each of tested individuals [control: 20; LH12: 15; LH12R1: 10; PS (control individuals injected with PS): 10; PVK 10^–9^ (individuals injected with PVK-2 in concentration 10^–9^ M): 10; PVK 10^–5^ (individuals injected with PVK-2 in concentration 10^–5^ M): 10].

The measurement of PO activity was supported by a semiquantitative reverse transcription–polymerase chain reaction (RT-PCR) analysis of the level of expression of the *proPO* gene (which encodes the inactive form of PO).

### Semiquantitative RT-PCR

Semiquantitative RT-PCR (sq-PCR) was performed according to a modification of the method described by Marone et al. (2001). This assay was used to analyze changes in the expression level of genes that encoding defensin (one of the burying beetle AMP) and proPO. The gene selection was based on the research conducted by Reavey et al. (2015) and Benowitz et al. (2019). Our choice was also verified using the data published by Cunningham et al. (2015) and Jacobs et al. (2016). Individuals in each of the research treatments were anesthetized with CO_2_ and then decapitated. Next, the elytra were removed, and the abdomen was cut. The abdomen without gut and reproductive organs was then transferred to 150 μL of RNA lysis buffer (Zymo Research, United States) and homogenized for 3 min using a pellet homogenizer. The homogenized tissues were immediately frozen in liquid nitrogen and then stored at −80°C. A Quick-RNA^®^ Mini Prep kit (Zymo Research, United States) was used for RNA isolation. After RNA isolation, DNase treatment of samples with Turbo DNase Kit (Thermo-Fisher Scientific, United States) was performed. The RNA concentration was determined using a Synergy H1 Hybrid Multi-Mode Microplate Reader (BioTek, United States). Reverse transcription of the same amount of isolated RNA (30 ng) to cDNA was accomplished using the RevertAid^TM^ Reverse Transcriptase kit (Thermo-Fisher Scientific, United States) according to the manufacturer’s protocol. Semi quantitative RT-PCR were conducted using a T100^TM^ Thermal Cycler (Bio-Rad, United States). The primers were designed based on sequences available in public databases (NCBI) using Primer3 software from the Geneious 9.1.8 suite. Briefly, the mRNA sequences of analyzed genes (*defensin*: XM_017915154.1; *proPO*: XM_017920422) retrieved from the NCBI database (all major available databases, including GenBank) were used as a template for primer design. Each primer pair was created to amplify fragments ranging in size from approximately 100 to 120 base pairs (bp). The primers were created by the Institute of Biochemistry and Biophysics of the Polish Academy of Science. The information about primer sequences and their location over exons can be found in the Supplementary Material (Supplementary Table 1 and Supplementary Figure 2). Moreover, Primer-BLAST analyses based on available genomic data from NCBI database (*N. vespilloides*, AN: PRJNA339573) exclude possibility of cross-amplification. On the other hand, defensin’s sequences published by Jacobs et al. (2016) may suggest potential amplification more than single gene (*defensin-1* and *defensin-2*). RT-PCR was performed in a 10-μL reaction volume containing 3.5 μL of DNase/RNase-free water, 1 μL of DreamTaq^TM^ Green Buffer (Thermo Fisher Scientific, United States), 1 μL of 2 mM dNTP, 1 μL of 10 μM forward primers, 1 μL of 10 μM reverse primers, 0.05 μL of DreamTaq^TM^ DNA polymerase (Thermo-Fisher Scientific, United States), and 2 μL of cDNA. The primers stocks (100 μM) were diluted with DNase/RNase-free water just before PCR reaction. The obtained products of RT-PCR were analyzed by electrophoresis using a 2.5% TAE agarose gel stained with ethidium bromide. The same volume of RT-PCR product was put on gel. The Quick-Load Purple 100 bp DNA Ladder (New England BioLabs, United States) was run on each gel. Photographs of the agarose gels were taken using ChemiDoc^TM^ Touch (Bio-Rad, United States). The ImageJ software was used to analyze the intensity of the bands. The results were expressed as the percent change in band intensity compared with the samples obtained from control individuals. To minimize the individual variation between insects, treatment groups were compared to the control samples performed on the same day. In each of the runs, two control samples were always loaded onto the gel. For checking potential foreign contamination of samples, “no template control” (DNA/RNA free water) and “no RT control” reactions were included in the analysis (Supplementary Figure 3). In each of the research treatments, a minimum of 5 biological repeats were made [control: 13; LH12: 5; LH12R1: 6; PS (control individuals injected with PS): 5; PVK 10^–9^ (individuals injected with PVK-2 in concentration 10^–9^ M): 6; PVK 10^–5^ (individuals injected with PVK-2 in concentration 10^–5^ M): 6. Moreover, obtained results are corrected based on the expression level of reference gene]. Based on research conducted by Cunningham et al. (2014), the *tbp* gene (gene encoding TATA-box-binding protein) was used as a reference (Supplementary Figure 4). The expression level of *tbp* in the experimental variants was compared to expression level of control samples. The obtained results were the correction factor for specific samples.

To confirm our results, the bands were sequenced by the Molecular Biology Techniques Laboratory (Faculty of Biology, Adam Mickiewicz University) and compared with data available in the public database (NCBI) (*N. vespilloides*, AN: PRJNA339573) using BLASTn protocol^2^. The PCR products were eluted from the agarose gel using Zymoclean Gel DNA Recover Kit (Zymo Research).

### Statistical Analysis

For statistical analysis, the GraphPad Prism 5 software was used (license: Department of Animal Physiology and Developmental Biology, Adam Mickiewicz University). The normality of data distribution was checked using Shapiro-Wilk test. For parametric data, one-way analysis of variance with Bonferroni *post hoc* test was used. Non-parametric data were tested using Mann–Whitney *U*-test and Kruskal-Wallis test with Dunn *post hoc* test.

## Results

### Weight Loss

The loss of weight by burying beetles during desiccation was statistically significant (Mann–Whitney *U*-test, *U* = 31.00, *p* ≤ 0.05) (Supplementary Figure 5). After 12-h low-humidity treatment, beetles were losing 18.81 ± 1.30% of their weight (mean ± SEM).

### Total Haemocyte Count

**FIGURE 1:**
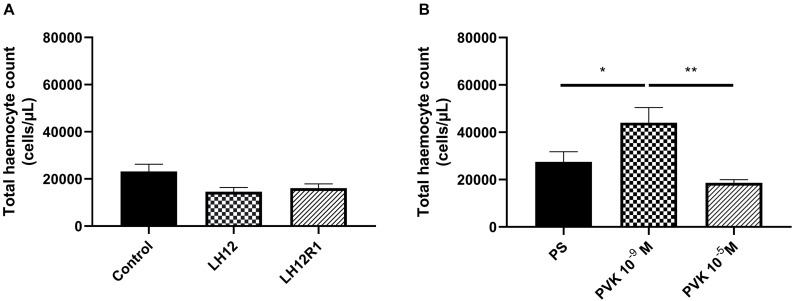
Total haemocyte count value after exposure of *N. vespilloides* to low humidity for 12 h (LH12), after 1-h recovery time (LH12R1) **(A)**, and 1 h after application of Tenmo–PVK-2 in concentration 10^–9^ and 10^–5^ M **(B)**. The values are presented as mean ± SEM, ^∗^*p* ≤ 0.05, ^∗∗^*p* ≤ 0.01.

The comparison of all tested groups showed statistically significant differences in THC value [one-way test, *F*_(5, 44)_ = 6.820; *p* ≤ 0.001]. However, after the exposure to low humidity and additional recovery time, the number of haemocytes did not change significantly (Figure 1A). On the other hand, an increase in the average number of haemocytes was observed after the injection of Tenmo–PVK-2 at a concentration 10^–9^ M (Bonferroni *post hoc*, *p* ≤ 0.05). These differences were statistically significant also in comparison to individuals injected with Tenmo–PVK-2 at a concentration 10^–5^ M (Bonferroni *post hoc*, *p* ≤ 0.01) (Figure 1B). It should also be mentioned that there were no statistical differences between control individuals and individuals injected with PS.

### Haemocyte Morphology and Their Surface Area

**FIGURE 2:**
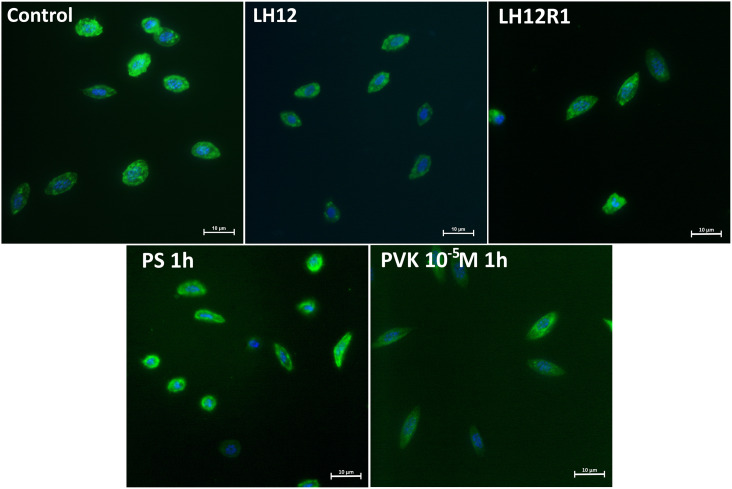
Representative fluorescent micrographs of *N. vespilloides* haemocytes after 12-h low-humidity treatment (LH12), 1 h of recovery (LH12R1), and 1 h after injection of physiological saline (PS) and Tenmo–PVK-2 at concentrations of 10^–5^ M. Green: F-actin cytoskeleton stained with Oregon Green^®^ phalloidin; blue: haemocyte nuclei visualized using DAPI solution.

**FIGURE 3:**
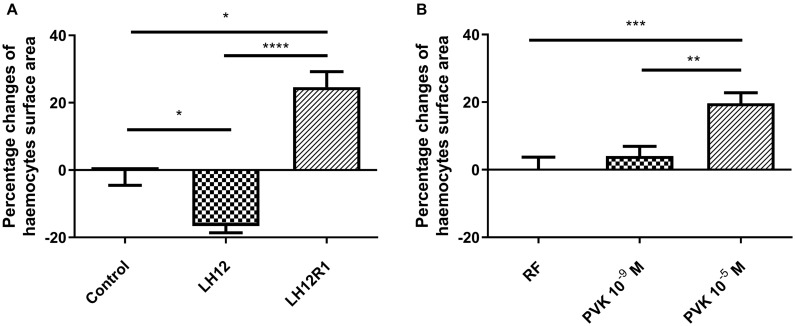
Percentage changes of haemocyte surface area of *N. vespilloides* compared to untreated individuals (control) or individuals injected with physiological saline (PS) **(A)** LH12: beetles exposed to low humidity for 12 h; LH12R1: beetles exposed to low humidity and recovered for 1 h; PVK 10^–9^ M and PVK 10^–5^ M: beetles analyzed 1 h after injection of Tenmo–PVK-2 at concentrations of 10^–9^ and 10^–5^ M, respectively **(B)**. Mean ± SEM, *p ≤ 0.05, **p ≤ 0.01, ***p ≤ 0.001, ****p ≤ 0.0001.

Both in LH12, LH12R1 groups and groups injected with different concentration of Tenmo–PVK-2, no disruptions of the F-actin cytoskeleton and nucleus were found (Figure 2). Only changes in the size of haemocytes were reported. These observations were supported by the results of the haemocyte surface area analysis (Figure 3). In the case of the LH12 group, a significant decrease in the surface area of haemocytes was noted (Dunn *post hoc*, *p* ≤ 0.05) (Figure 3A). On the other hand, 1-h recovery after low-humidity treatment caused a significant increase in the surface area of the haemocytes (Dunn *post hoc*, *p* ≤ 0.05). In case of research variants with the injection of Tenmo–PVK-2, neuropeptide application led to an increase in the surface area of the haemocytes. It was especially visible at a concentrations of 10^–5^ M (Dunn *post hoc*, *p* ≤ 0.001) (Figure 3B).

### Haemocyte Fractions

As in the previous study, three different types of haemocytes were distinguished: plasmatocytes, granulocytes, and prohaemocytes (Supplementary Figure 6). The differences between groups in percentage ratio of each of the observed haemocyte types were not observed (Supplementary Figure 7). Plasmatocytes were the main group of haemocytes (mean ± SEM, 59.38 ± 2.66%). The second numerous groups were granulocytes (mean ± SEM, 35.96 ± 2.52%), whereas only 4.65 ± 0.48% (mean ± SEM) of haemocytes were identified as prohaemocytes.

### Percentage Ratio of Phagocytosis

**FIGURE 4:**
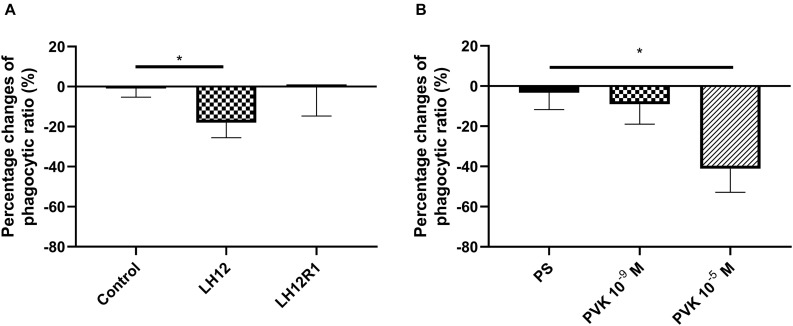
Percent change in the proportion of *N. vespilloides* haemocytes involved in phagocytosis after 12 h of low-humidity treatment (LH12), low humidity and recovery time (LH12R1) **(A)**, and 1 h after administration of Tenmo–PVK-2 at concentrations of 10^–9^ and 10^–5^ M **(B)**. Control: untreated beetles; PS: beetles analyzed 1 h after injection of physiological saline for beetles. Value are means ± SEM, *p ≤ 0.05.

The number of haemocytes participating in phagocytosis changed after exposure to low humidity and after administration of Tenmo–PVK-2 (Figure 4). In the case of LH12, a significant decrease in the number of phagocytic haemocytes was observed (Dunn *post hoc*, *p* ≤ 0.05). Interestingly, after the recovery time, this effect was not reported, and the phagocytic ratio returned to the control level (Figure 4A). A similar effect to low humidity was evoked by the injection of PVK, but only at the concentrations of 10^–5^ M. In this research variant, significant reduction in the number of phagocytic haemocytes was observed (Bonferroni *post hoc*, *p* ≤ 0.05) (Figure 4B).

### Phenoloxidase Activity and Expression Level of *proPO*

**FIGURE 5:**
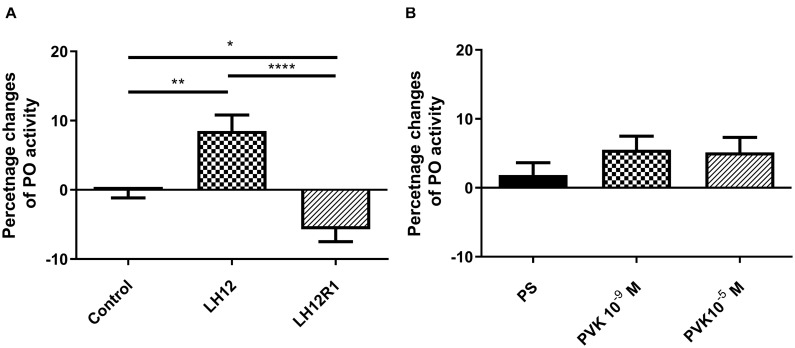
Changes of phenoloxidase activity in the hemolymph of *N. vespilloides* compared to untreated individuals (control) or beetles injected with physiological saline (PS). **(A)** LH12: beetles exposed to low humidity for 12 h and LH12R1: beetles exposed to low humidity and recovered for 1 h; PVK 10^–9^ M and PVK 10^–5^ M: beetles analyzed 1 h after injection of Tenmo–PVK-2 at concentrations of 10^–9^ and 10^–5^ M **(B)**. The data are presented as mean ± SEM, *p ≤ 0.05, **p ≤ 0.01, ****p ≤ 0.0001.

The level of PO activity in burying beetle hemolymph changes significantly especially in the case of the LH12 and LH12R1 groups (Figure 5). Compared to control individuals, the activity of PO increased significantly after 12-h exposure to low humidity (Bonferroni *post hoc*, *p* ≤ 0.01). Interestingly, after 1 h of recovery, compared to control, a statistically significant decrease in PO activity was noted (Bonferroni *post hoc*, *p* ≤ 0.05) (Figure 5A). In the case of injection of Tenmo–PVK-2, no significant differences were observed (Figure 5B).

**FIGURE 6:**
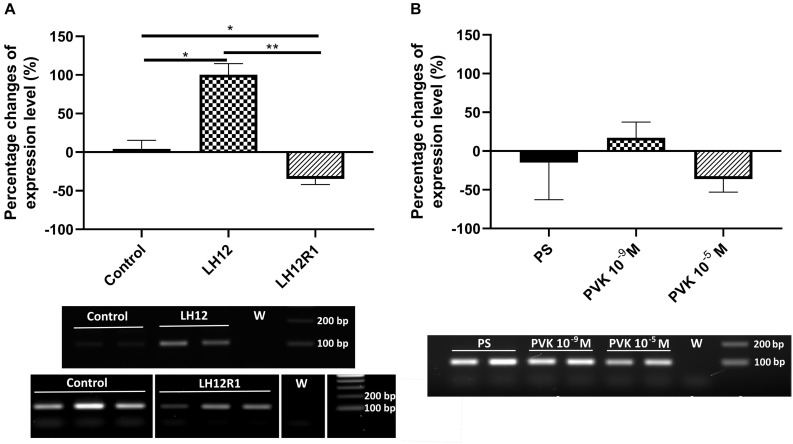
Semiquantitative analysis of the expression level of gene encoding proPO, precursor for phenoloxidase in the *N. vespilloides* body. The samples were collected 12 h after exposition to low humidity (LH12R), 1-h recovery time (LH12R1) **(A)**, and 1 h after injection of one of the CAPA–PVK neuropeptide, Tenmo–PVK-2 at concentrations of 10^–9^ and 10^–5^ M **(B)**. Below are exemplary results of the semiquantitative RT-PCR analyses. The values are presented as mean ± SEM, *p ≤ 0.05, **p ≤ 0.01.

The results of sq-RT-PCR analysis of expression level of *proPO* are consistent with the results of the PO activity (Figure 6). As in the enzymatic assay, compared to control, there was a significant increase in the level of *proPO* expression in the LH12 group (Bonferroni *post hoc*, *p* ≤ 0.05). Moreover, in LH12R1, the expression level of *proPO* was significantly decreased in comparison to non-treated individuals (Bonferroni *post hoc*, *p* ≤ 0.05) (Figure 6A). Similarly to the PO assay, no significant differences were observed in the Tenmo–PVK-2–injected group (Figure 6B).

### Expression Level of *defensin*

**FIGURE 7:**
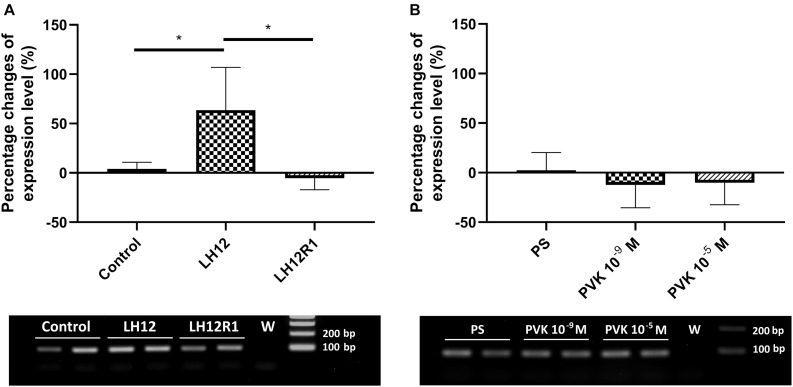
Percent changes in expression of the gene encoding defensin in *N. vespilloides* after exposure to stressful conditions and after administration of CAPA–PVK neuropeptide, compared to control individuals. **(A)** LH12: beetles exposed to low humidity for 12 h and LH12R1: beetles exposed to low humidity and recovered for 1 h; PVK 10^–9^ M and PVK 10^–5^ M: beetles analyzed 1 h after injection of Tenmo–PVK-2 at concentrations of 10^–9^ and 10^–5^ M **(B)**. Below are exemplary results of the semiquantitative RT-PCR analyses. The data presented as mean ± SEM, *p ≤ 0.05.

The expression level of *defensin* also changes significantly, but only with low-humidity treatment (Kruskal-Wallis test, *H* = 9.32, *p* ≤ 0.01) (Figure 7). Compared to control, in the LH12 group, a statistically significant increase in the level of *defensin* expression was observed (Dunn *post hoc*, *p* ≤ 0.05). For the other tested groups, the changes were insignificant.

## Discussion

Here we report the effect of short-time desiccation and recovery time on the functioning of various immune mechanisms of the burying beetle *N. vespilloides*. Because of better understanding of observed changes in the activity of immune mechanisms, especially those reported during the recovery time, the effects of Tenmo–PVK-2 were also examined in the presented study. Generally, some similarities were observed between the individuals after recovery time after low-humidity treatment and after injection of Tenmo–PVK-2, especially for the cellular response. Moreover, to our knowledge, the presented studies are the first evidence of the influence of CAPA–PVK neuropeptides on this type of insect immune response.

Interestingly, THC value did not change almost in all research variants. In the case of low-humidity treatment and after recovery time, only a slight insignificant decrease in the number of haemocytes was noted. However, it should be mentioned that the THC value estimates the number of haemocytes in 1 μL of hemolymph. In case of low-humidity treatment, the decrease in hemolymph volume was observed (personal communication). Because of these, we did not exclude that decrease in hemolymph volume may be related to decrease in the number of all haemocytes circulating in the hemolymph.

On the other hand, after application of PVK, the number of observed cells in the burying beetles increased significantly, but only at a concentration 10^–9^ M. These results may suggest that, like octopamine (OCT), CAPA–PVK neuropeptides can stimulate the release of haemocytes from hemopoietic organs (Adamo, 2010). However, the higher concentration did not elicit any effect, which may indicate a dose-dependent action of CAPA–PVK neuropeptide.

The results of the assessment of haemocytes showed that these cells react differently to the low-humidity treatment and during recovery time. Because of the fact that the adhesive ability of cells can be an indicator of membrane composition (Sun et al., 2007; Zeisig et al., 2007), these changes are likely related to remodeling of the cell membrane, especially with regard to phospholipid composition. Decrease in surface area during the desiccation is likely to be associated with a reduction in the membrane’s phospholipid-derived fatty acid–unsaturation level and a decrease in membrane fluidity and water permeability (Kieft et al., 1994; Holmstrup et al., 2002). However, for the confirmation of this supposition, further studies are needed.

On the other hand, during the recovery time and after the injection of Tenmo–PVK-2, a significant increase in the surface area of haemocytes was observed, which may indicate a greater adhesive ability. These results are consistent with the action of NMU on immune cells. Research by Moriyama et al. (2006) has shown that NMU increases the adhesion ability of eosinophils. Because of these, there is a possibility that observed increase in the surface area of haemocytes after the recovery period is correlated with the release of CAPA–PVK neuropeptides (Terhzaz et al., 2015). However, the strict physiological role of increasing the adhesion ability during recovery is unknown. Also, to our knowledge, the presence of CAPA–PVK receptor on haemocyte surface area is not confirmed, which may suggest that observed effects are indirect and may be related to influence of tested neuropeptide on regulation of water balance. On the other hand, also we did not also exclude that the CAPA–PVK receptors are present on haemocytes and the observed effects are the results of direct action of PVK. For these reasons, further research is needed.

The results of the adhesion ability of haemocytes are partly related to the results of the phagocytic assay. Suppression of phagocytosis was observed under the difficult environmental conditions, associated with prolonged low humidity. This effect can be related to the likely lower fluidity of the haemocyte membrane. This supposition is supported by the results of the phagocytic assay after the recovery time, because the number of haemocytes participating in this cellular mechanism returns to the control level. With Tenmo–PVK-2 injection, a decrease in the phagocytic ratio was observed. These changes are probably related to the increasing adhesion ability of haemocytes. As shown by the results obtained by Valerius et al. (1981) and Urbański et al. (2018), greater cell adhesion may lead to decreased chemotaxis and phagocytosis of cells.

The differences in the activity of humoral mechanisms are also observed between the experimental treatments. However, all observed changes were noted only in the case of lower-humidity treatment and recovery time. These results suggested that CAPA–PVK neuropeptides, like NMU, mainly influence the cellular response. In the case of PO, the low-humidity treatment led to an increase in the activity of the tested enzyme. Also, in this group, there were significant increases in the expression level of the gene encoding proPO. PO plays a multifunctional role in insects. This enzyme is responsible for the activation of melanogenesis, which is also important for insect immune system functioning (González-Santoyo and Córdoba-Aguilar, 2012). Because of this, observed changes in the PO activity and the expression level of *proPO* probably are connected with participation of this enzyme in melanin deposition than its immune action. This is related to the fact that melanins are a potential barrier to the movement of water across the cuticle (Parkash et al., 2008). This supposition supports the results observed in the treatment with an extra recovery period, as in these individuals a decrease in the activity of PO and the level of *proPO* expression were observed. This may suggest that the insects have reduced the melanin content in the cuticle during intense rehydration.

Significant changes in the expression of defensin-encoding genes have been also noted. However, the elevated level of *defensin* expression was observed only during low-humidity treatment. These results were expected because many genomic analyses show up-regulation of immune-related genes under stressful environmental conditions (Brown et al., 2009; Zhang et al., 2011; Salehipour-Shirazi et al., 2017). One of the proposed hypotheses is that insects developed a similar response to harsh environmental conditions and pathogen infection (cross-talk) (Sinclair et al., 2013). However, we should not exclude that enhanced expression level of *defensin* is also related to an increase in heat shock proteins and ferritin concentration during exposition to low humidity or stress-induced injuries (Yi and Lee, 2003; Clark and Worland, 2008; Linder et al., 2008; Tusong et al., 2017; Wojda, 2017).

Lack of differences in the *defensin* expression level after PVK injection may suggest that CAPA–PVK neuropeptides did not influence on the regulation of AMPs synthesis. This result is consistent to the results obtained by Terhzaz et al. (2014), which clearly showed that application of Drome–CAPA-1 did not lead to the expression of the gene that encodes diptericin in *D. melanogaster*.

Also, it should be mentioned that used sq-PCR technique has some limitations, especially related to it sensitivity. For this reason, some of the effects associated with stress treatment and CAPA–PVK injection may be not fully exposed, and further studies concerning this issue are required. However, as presented by Marone et al. (2001) and Breljak et al. (2005), sq-PCR, although laborious and time consuming, may remain useful techniques for relative mRNA quantification when a small number of samples are to be analyzed.

In summary, in the presented research, the effect of low humidity on the immune system of burying beetles was tested. The results obtained partially support several hypotheses concerning the morphological and physiological adjustments that are observed during the insect organism’s response to environmental stress, including changes in the ability of haemocytes to adhesion or potential melanin deposition, which prevents water loss. These results suggest that some changes in immune system functioning during stress conditions do not have an immune function. Interestingly, part of the effects characteristic of recovery time were also observed after the application of Tenmo–PVK-2, mainly related to haemocyte morphology, which indicate that CAPA–PVK neuropeptides may also participate in modulation of insect immune response. However, the mode of action of CAPA–PVK neuropeptides on cellular response is still unknown, and further studies are needed.

## Data Availability Statement

The raw data supporting the conclusions of this article will be made available by the authors, without undue reservation.

## Author Contributions

AU: design of the work, maintain of insect culture, performance of bioassays, analysis, and interpretation of the results, the manuscript preparation. KW-N: performance of bioassays, analysis, and interpretation of the results, the manuscript edition. GN: analysis and interpretation of the results, the manuscript edition. SC: samples collection and the manuscript edition. GR: manuscript editing and substantive support. All authors contributed to the article and approved the submitted version.

## Conflict of Interest

AU is employed by the company HiProMine S.A. GN is employed by the company genXone S.A. The remaining authors declare that the research was conducted in the absence of any commercial or financial relationships that could be construed as a potential conflict of interest.

## Footnotes

1 Neuropeptide nomenclature based on the Coast and Schooley (2011).

2 https://blast.ncbi.nlm.nih.gov/Blast.cgi
