# Supplementary material for: Effect of Short-Term Desiccation, Recovery Time, and CAPA–PVK Neuropeptide on the Immune System of the Burying Beetle Nicrophorus vespilloides
Source: Front Physiol. 2021 Jun 21;12:671463. doi: 10.3389/fphys.2021.671463 (PMC8255627; doi:10.3389/fphys.2021.671463)
Supplement: Supplementary file 1 [file Data_Sheet_1.docx]

**Raw photos of agarose gels with exemplary results of the semi–quantitative RT-PCR analyses presented in: Effect of short-term desiccation, recovery time and CAPA-PVK peptides on the immune system of the burying beetle *Nicrophorus vespilloides***

Urbański A.^1,2^, Walkowiak-Nowicka K.^1^, Nowicki G.^3,4^, Chowański S.^1^, Rosiński G.^1^

^1^Department of Animal Physiology and Developmental Biology, Faculty of Biology, Adam Mickiewicz University in Poznań, Poland;

^2^HiProMine S.A., Robakowo, Poland;

^3^Molecular Virology Research Unit, Faculty of Biology, Adam Mickiewicz University in Poznań, Poland

**
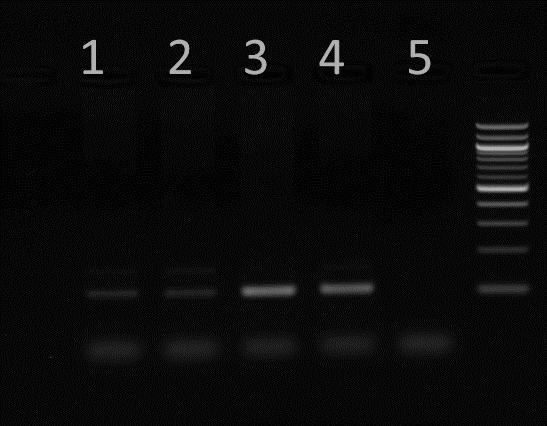
**^4^genXone S.A., Kobaltowa 6 Str., Złotniki, Poland

**Fig.** Changes of expression level of the gene encoding proPO in *N. vespilloides*; 1 and 2 - Control individuals; 3 and 4 –beetles exposed to low humidity for 12 h; 5 – negative control (water).

**
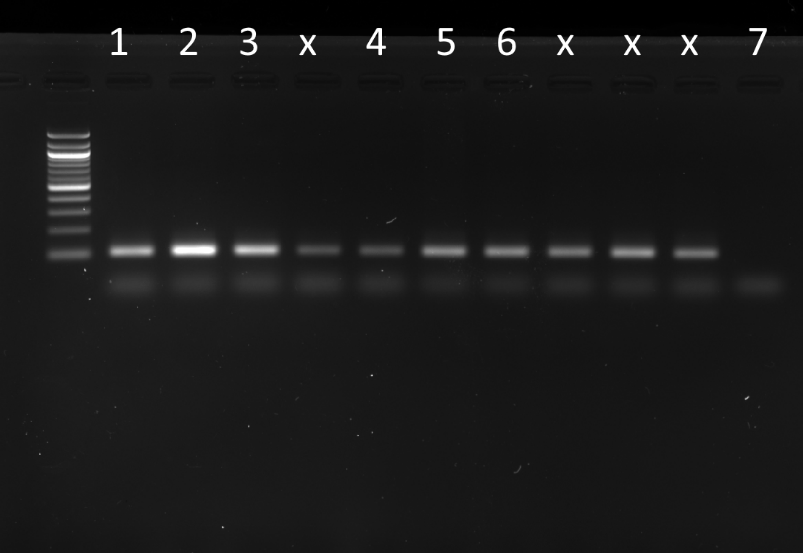
**

**Fig.** Changes of expression level of the gene encoding proPO in *N. vespilloides*; 1, 2 and 3 - Control individuals; 4, 5 and 6 –beetles exposed to low humidity for 12 h and extra 1h recovery time; 7 – negative control (water); During the manuscript preparation, bands order was changed and the line with marker was replaced into the left. X – bands not shown in the Figure 6.

**
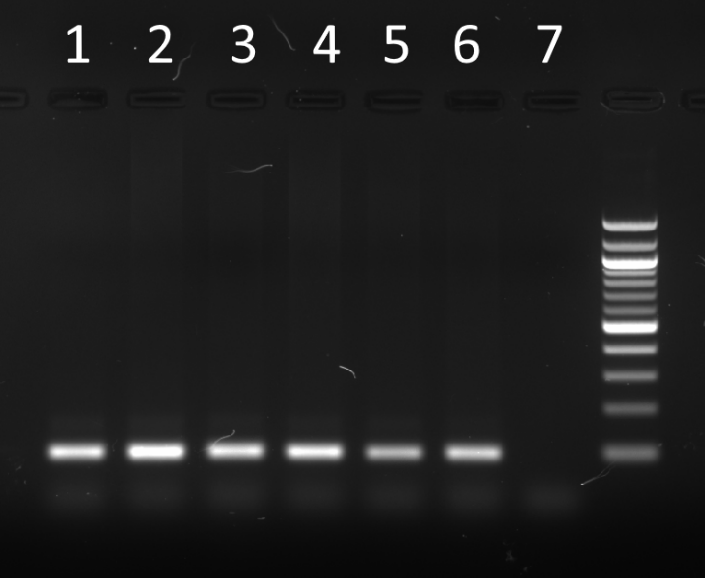
**

**Fig.** Changes of expression level of the gene encoding proPO in *N. vespilloides*; 1 and - Control individuals injected with physiological saline; 3 and 4 – beetles injected with Tenmo-PVK at the concentration 10^-9^ M, 5 and 6 –beetles injected with tested neuropeptide at concentration 10^-5^ M; 7 – negative control (water).

**
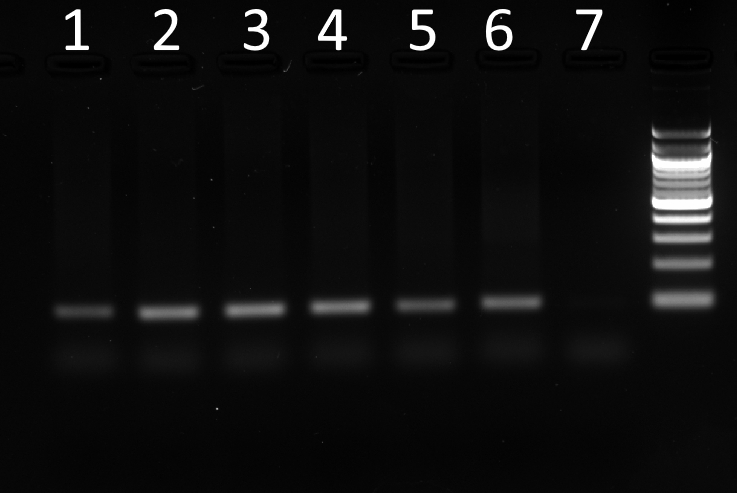
**

**Fig.** Changes of expression level of *defensin* gene encoding in *N. vespilloides*; 1 and - Control individuals; 3 and 4 – beetles treated with low humidity for 12 h, 5 and 6 –samples collected from beetles after 12 h of low humidity treatment and 1 h of recovery time; 7 – negative control (water).

**
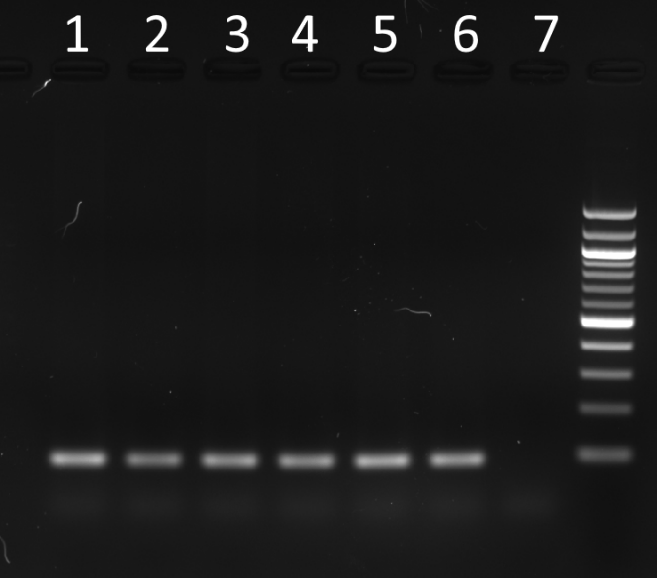
**

**Fig.** Changes of expression level of *defensin* gene in *N. vespilloides*; 1 and - Control individuals injected with physiological saline; 3 and 4 – beetles injected with Tenmo-PVK at the concentration 10^-9^ M, 5 and 6 –beetles injected with tested neuropeptide at concentration 10^-5^ M; 7 – negative control (water).
