# Supplementary material for: Effect of Short-Term Desiccation, Recovery Time, and CAPA–PVK Neuropeptide on the Immune System of the Burying Beetle Nicrophorus vespilloides
Source: Front Physiol. 2021 Jun 21;12:671463. doi: 10.3389/fphys.2021.671463 (PMC8255627; doi:10.3389/fphys.2021.671463)
Supplement: Supplementary file 2 [file Data_Sheet_2.doc]

**Supplementary materials for: Effect of short-term desiccation, recovery time and CAPA-PVK peptides on the immune system of the burying beetle *Nicrophorus vespilloides***

Urbański A.^1,2^, Walkowiak-Nowicka K.^1^, Nowicki G.^3,4^, Chowański S.^1^, Rosiński G.^1^

^1^Department of Animal Physiology and Developmental Biology, Faculty of Biology, Adam Mickiewicz University in Poznań, Poland;

^2^HiProMine S.A., Robakowo, Poland;

^3^Molecular Virology Research Unit, Faculty of Biology, Adam Mickiewicz University in Poznań, Poland

^4^genXone S.A., Kobaltowa 6 Str., Złotniki, Poland


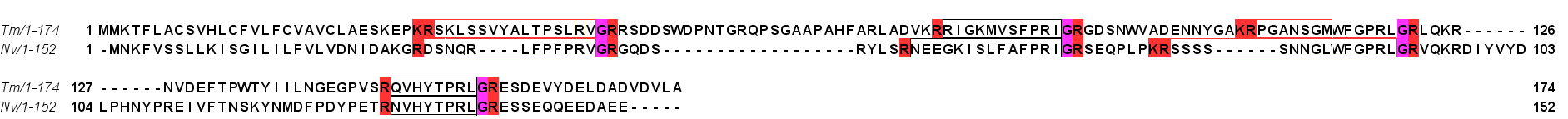
**Tab. S1.** Primers used in the presented study.

**Fig. S1.** Alignment of the CAPA-PVK precursor sequences from *Tenebrio molitor* and *Nicrophorus vespilloides* (AN: XP_017773240). Pink - amidation sites; Red - endopeptidase cleavage sites; red frames indicate the deduced CAPA/PVK isoforms.


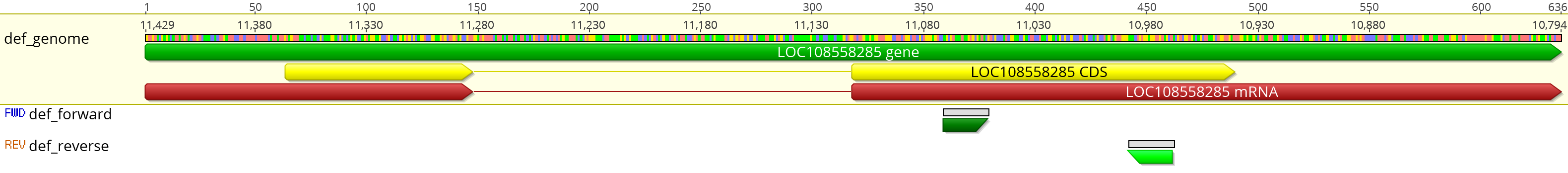

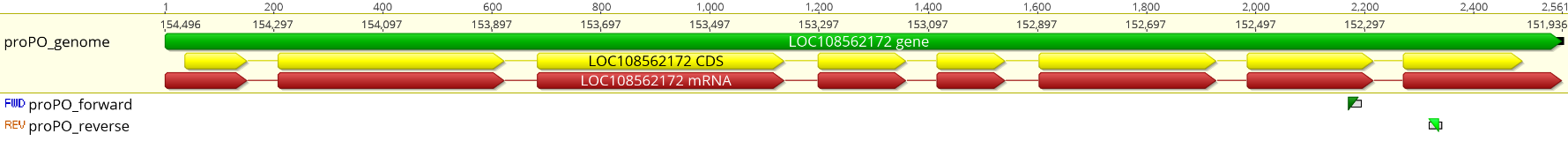


**Fig. S2.** Exon-intron structure of *defensin* (XM_017915154.1) and *proPO* (XM_017920422) with schematic presentation of localization of used primers.


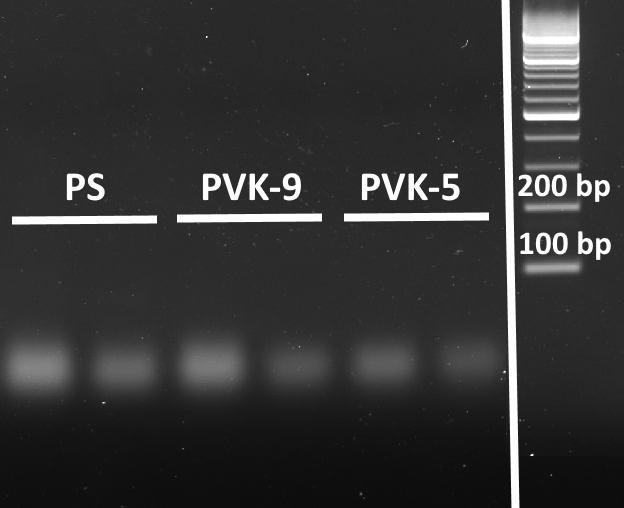

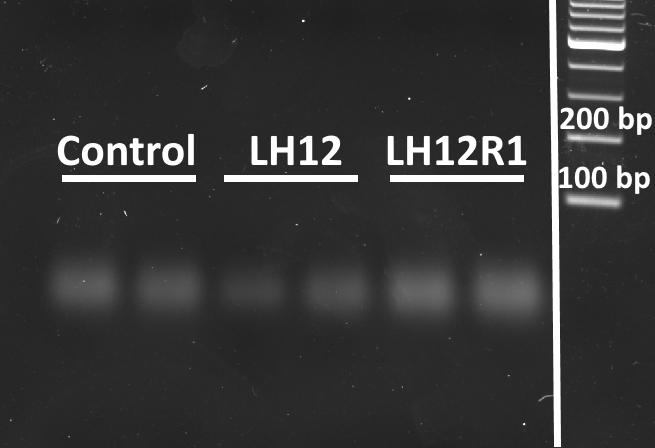


**Fig. S3.** Negative control for RT-PCR analysis, without using of reverse transcriptase during transcription of RNA to cDNA; Control, non-treated individuals; LH12, exposure to low humidity for 12 h; LH12R1 – exposure to low humidity LH12 after 1h recovery time recovery time; PS - beetles injected with physiological saline 1 h before analysis; PVK 10^-9^ M and PVK 10^-5^ M – beetles injected with solution of physiological saline and Tenmo-PVK-2 in concentration 10^-9^ and 10^-5^ M.

**
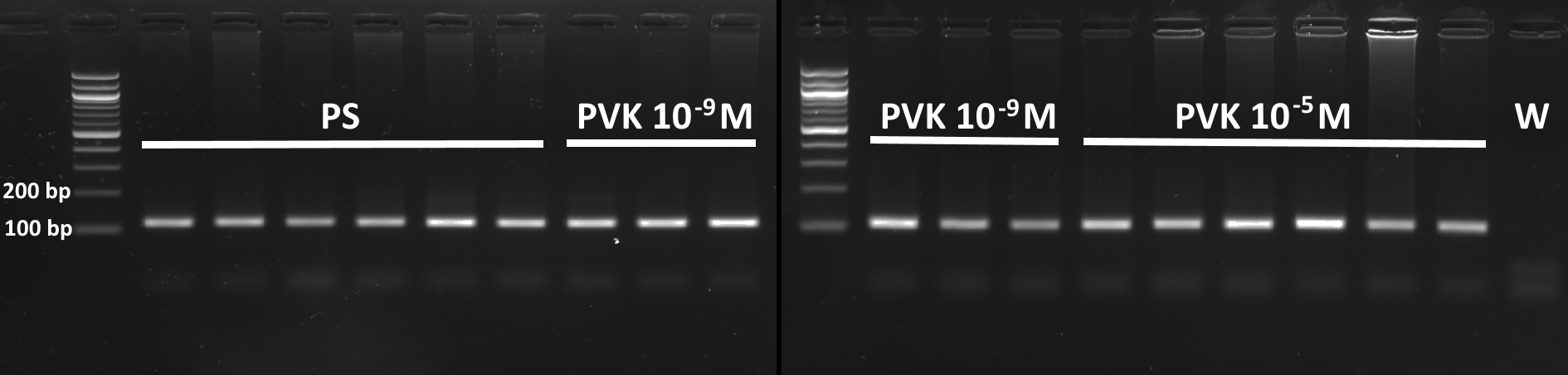

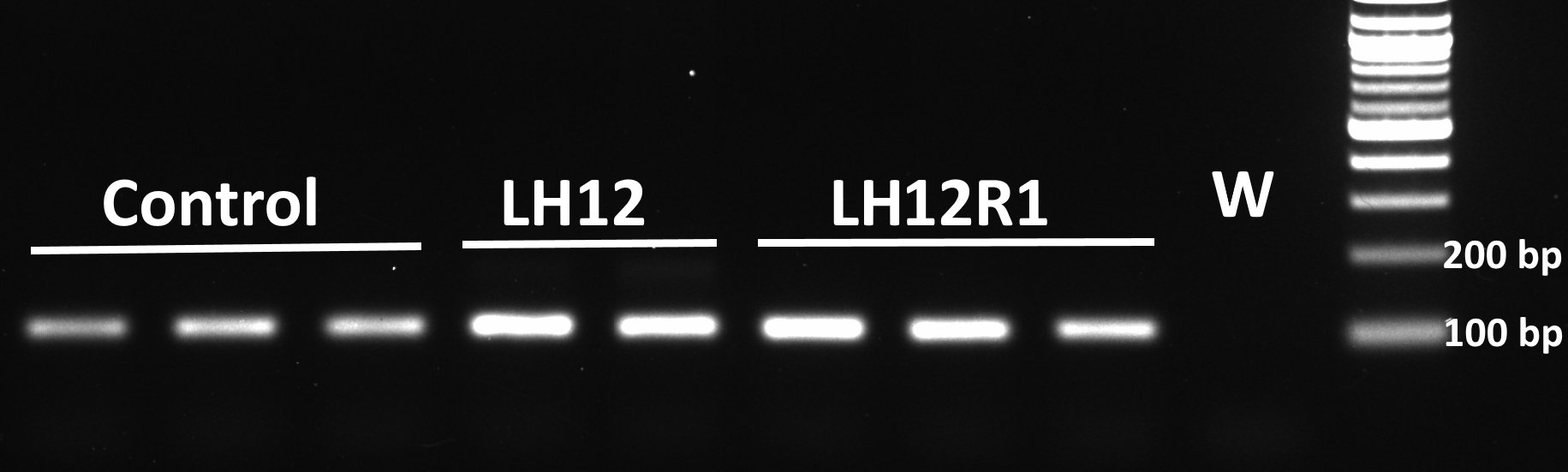
**

**Fig. S4.** Representative results of the semi-quantitative RT-PCR analyses for the tbp gene in *N. vespilloides*. Electrophoresis of RT-PCR products was performed using a 2.5% TAE agarose gel stained with ethidium bromide. Control, control individuals; LH12, exposure to low humidity for 12 h; LH12R1 – exposure to low humidity LH12 after 1h recovery time recovery time; PS - beetles injected with physiological saline 1 h before analysis; PVK 10^-9^ M and PVK 10^-5^ M – beetles injected with solution of physiological saline and Tenmo-PVK-2 in concentration 10^-9^ and 10^-5^ M. For sq-RT-PCR a minimum of 5 biological and 3 technical repeats were made.


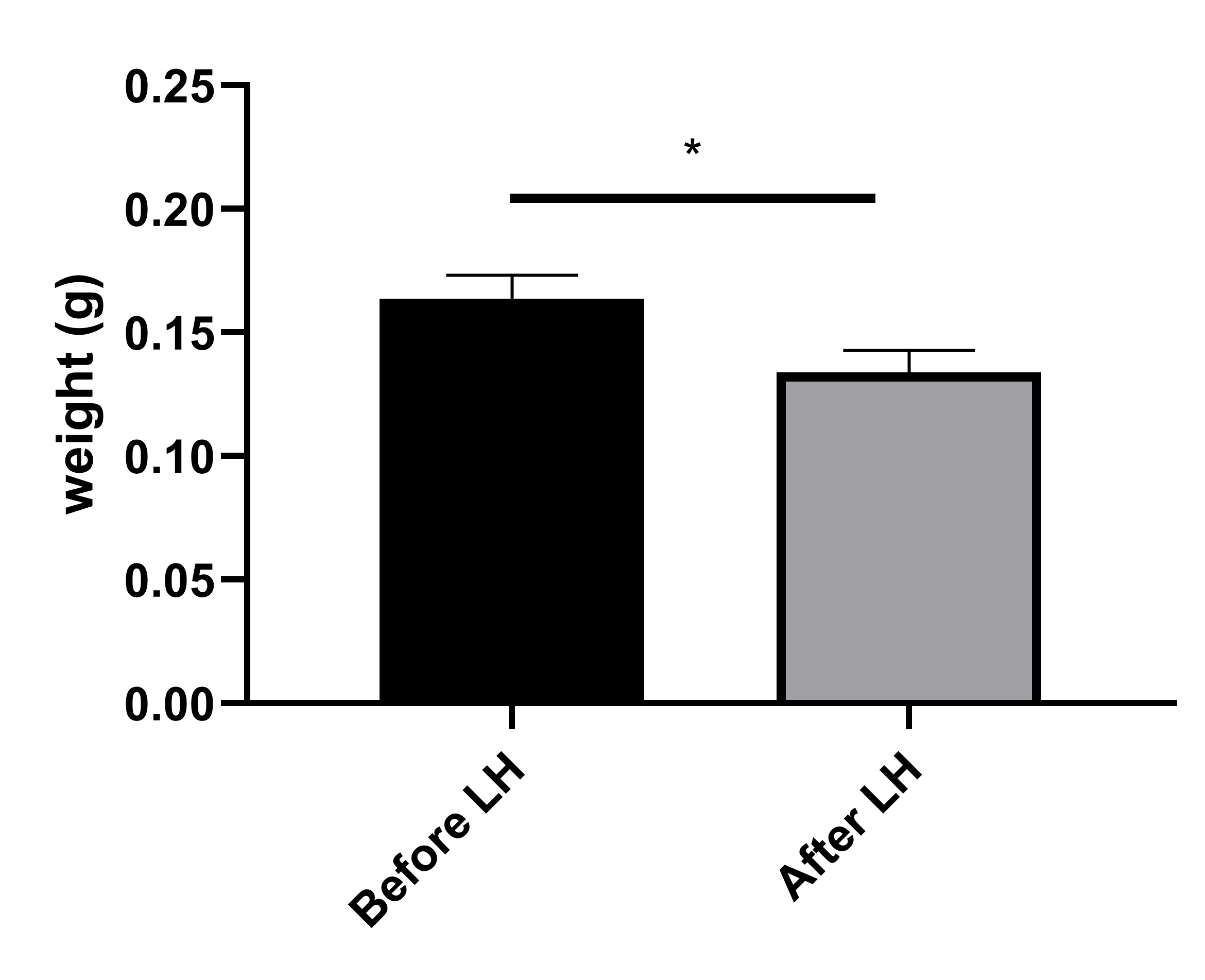
**Fig S5**. Changes in weight of burying beetles *N. vespilloides* after 12 h low humidity treatment. LH – 12 h low humidity treatment. Mean±SEM; * *p*≤0.05.


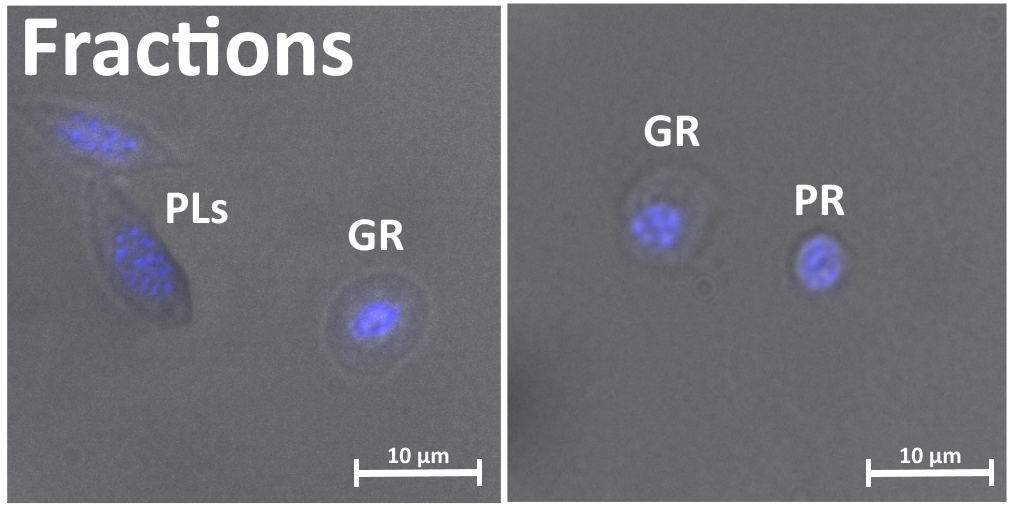


**Fig. S6.** Representative micrographs presenting the haemocyte’s types observed in the burying beetles *N. vespilloides*. Blue – nucleic acids stained with DAPI solution. PLs – plasmatocytes; GR – granulocyte: PR – prohaemocyte.


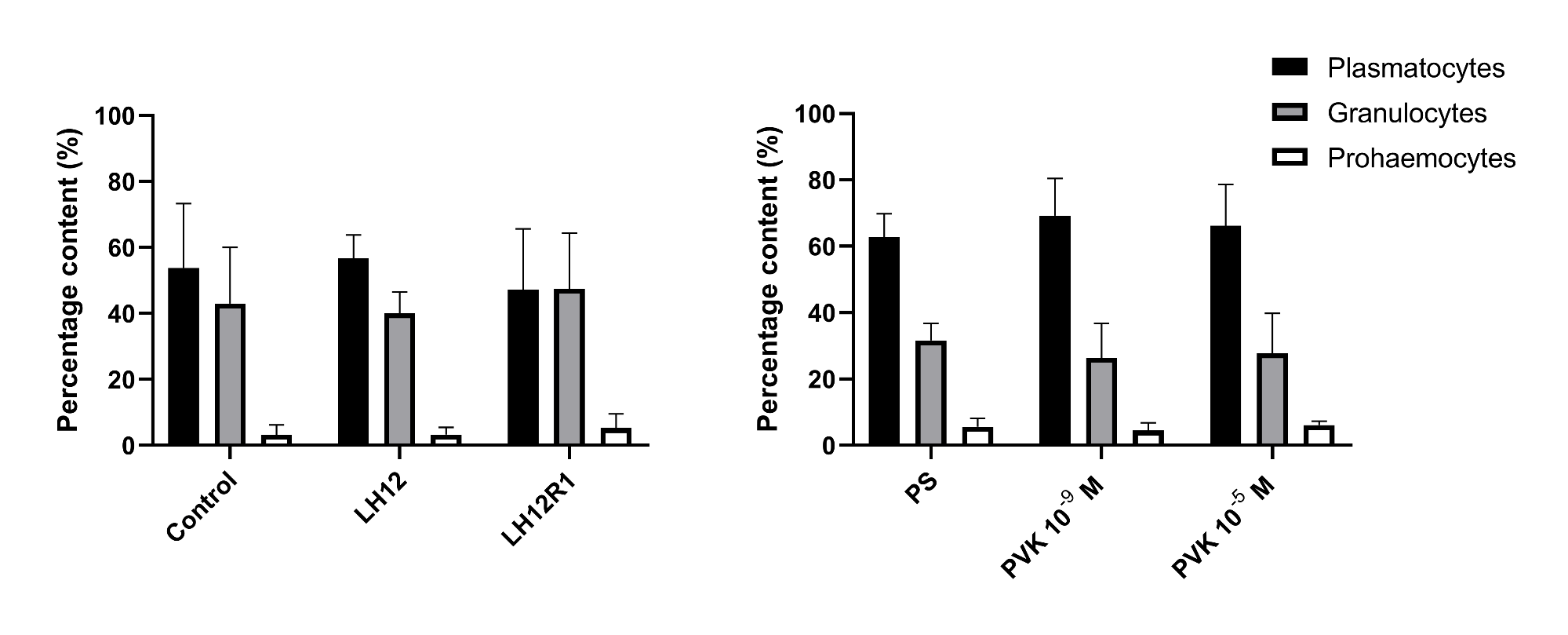


**Fig. S7.** Percentage proportion of haemocyte’s type in the haemolymph of *N. vespilloides* after 12 h exposition to low humidity (LH12), recovery time (LH12R1) and 1 h after application of Tenmo-PVK-2 in concentration 10^-9^ and 10^-5^ M. Control – untreated individuals; PS – beetles injected with physiological saline 1 h before analysis. Mean±SEM.

**References**

Neupert, S., Marciniak, P., Koehler, R., Nachman, R. J., Suh, C. P. C., & Predel, R. (2018). Different processing of CAPA and pyrokinin precursors in the giant mealworm beetle *Zophobas atratus* (Tenebrionidae) and the boll weevil *Anthonomus grandis grandis* (Curculionidae). *Gen Comp Endocrinol*, 258, 53-59.
